# Validity and usability testing of a health systems guidance appraisal tool, the AGREE-HS

**DOI:** 10.1186/s12961-018-0334-9

**Published:** 2018-06-20

**Authors:** Melissa C. Brouwers, Denis Ako-Arrey, Karen Spithoff, Marija Vukmirovic, Ivan D. Florez, John N. Lavis, Francoise Cluzeau, Govin Permanand, Xavier Bosch-Capblanch, Yaolong Chen, On behalf of the AGREE-HS Research Team, Andy Haines, Carmen Mihaela Dolea, Fadi El-Jardali, Jillian Ross, Luis Gabriel Cuervo, Mike Wilson, Mita Giacomini, Pablo Perel, Padraig Warde, Pierre Ongolo-Zogo, Sheila McNair, Ulysses Panisset

**Affiliations:** 1 McMaster University, Juravinski Site, G2 Wing, 1280 Main Street West, Hamilton, ON L8S 4L8 Canada; 2 McMaster University, 1280 Main Street West MML-417, Hamilton, ON L8S 4L8 Canada; 3 Imperial College London, St. Mary’s Hospital (Room 1070, Queen Elizabeth the Mother Wing), Praed Street, London, W2 1NY United Kingdom; 4 The London School of Economics and Political Science, Houghton Street, London, WC2A 2AE United Kingdom; 5 Swiss Tropical and Public Health Institute, Socinstrasse 57, 4002 Basel, Switzerland; 6 Universität Basel, Petersplatz 1, 4003 Basel, Switzerland; 7 Lanzhou University (Evidence Based Medicine Center), 222 Tianshui S Rd, Chengguan Qu, Lanzhou Shi, Gansu Sheng China

**Keywords:** Health systems guidance, Health system, Health policy, Quality appraisal

## Abstract

**Background:**

Health systems guidance (HSG) provides recommendations to address health systems challenges. No tools exist to inform HSG developers and users about the components of high quality HSG and to differentiate between HSG of varying quality. In response, we developed a tool to assist with the development, reporting and appraisal of HSG – the Appraisal of Guidelines for Research and Evaluation–Health Systems (AGREE-HS). This paper reports on the validity, usability and initial measurement properties of the AGREE-HS.

**Methods:**

To establish face validity (Study 1), stakeholders completed a survey about the AGREE-HS and provided feedback on its content and structure. Revisions to the tool were made in response. To establish usability (Study 2), the revised tool was applied to 85 HSG documents and the appraisers provided feedback about their experiences via an online survey. An initial test of the revised tool’s measurement properties, including internal consistency, inter-rater reliability and criterion validity, was conducted. Additional revisions to the tool were made in response.

**Results:**

In Study 1, the AGREE-HS Overview, User Manual, quality item content and structure, and overall assessment questions were rated favourably. Participants indicated that the AGREE-HS would be useful, feasible to use, and that they would apply it in their context. In Study 2, participants indicated that the quality items were easy to understand and apply, and the User Manual, usefulness and usability of the tool were rated favourably. Study 2 participants also indicated intentions to use the AGREE-HS.

**Conclusions:**

The AGREE-HS comprises a User Manual, five quality items and two overall assessment questions. It is available at agreetrust.org.

**Electronic supplementary material:**

The online version of this article (10.1186/s12961-018-0334-9) contains supplementary material, which is available to authorized users.

## Background

Health systems guidance (HSG) provides systematically developed recommendations to manage challenges related to health system governance, financial and delivery arrangements, and the implementation strategies needed to get the right programmes and services to those who need them [1–3]. Further, HSG aims to assist in decision-making, particularly decisions made by health policy-makers, health services managers, healthcare providers, and system and institutional leaders [1–3].

There is concern about the quality of HSG documents that are currently available. These concerns stem from the recognition of the overall complexity of health systems and the ability to develop timely, contextually relevant recommendations; the use of the broad range of different types of evidence needed to develop HSG (e.g. evidence generated outside of the traditional research paradigm); the unique factors that may influence the design and adoption of recommended actions; and the general lack of experience and expertise in working with this evidence in a systematic and transparent manner [1, 4–7]. The field of HSG is relatively new; thus, unlike other guidance fields, such as clinical practice guidelines (CPGs), there has not yet been enough time to fully cultivate the best methods for HSG development, reporting and appraisal [8]. Nonetheless, the risks of varying quality HSG are not inconsequential. The use of poor quality HSG to inform decision-making may result in the adoption of health system arrangements and implementation strategies that are contextually inappropriate or ineffective, if not harmful and extremely costly; this may put populations at harm, waste precious resources, and undermine the credibility of, and confidence in, future health system improvement efforts [1, 7].

To help mitigate these risks, an international consortium of researchers, the Appraisal of Guidelines for Research and Evaluation–Health Systems (AGREE-HS) Research Team, has undertaken a programme of research aimed at creating a tool to support the development, reporting and appraisal of HSG [9]. A critical interpretive synthesis of the literature was conducted to identify concepts related to HSG quality that could serve as criteria for an HSG appraisal tool [10]. Thirty candidate concepts were identified and all were judged to be important and comprehensive by a sample of international HSG developers, users (e.g. policy-makers and a broad range of stakeholders including managerial, professional and patient groups) and researchers; an additional two concepts were recommended for inclusion [10]. Using these data, a prototype tool was designed. The 32 concepts were converted into an item format and linked to one of four domains. Each of the items had an operational definition, instructions for use, and the proposed response scale. A second international sample of participants provided feedback on the prototype tool and its application [11]. Respondents provided favourable ratings about the tool’s ease of application and usefulness; however, they also offered several suggestions to improve the tool, including changing the response scale [11]. Moreover, there was recognition by the Research Team that the application of a 32-item tool would likely be too time-consuming to make it feasible for most users and that a 7-point response scale, while preferred, would not be appropriate for many of the items, given their specificity.

In response, the tool was refined to create the AGREE-HS Draft, consisting of five core quality items (topic, participants, methods, recommendations and implementability). The original 32 items were reframed as criteria and categorised into one of these five quality items. The purpose of this article is to describe two studies designed to test the tool.

## Methods

### Overview

In Study 1, participants reviewed and systematically assessed the AGREE-HS Draft, without applying it. In Study 2, participants used a revised version of the AGREE-HS Draft (i.e. the AGREE-HS Final Draft) to appraise a collection of HSG documents; they subsequently reflected on their experiences in applying the AGREE-HS Final Draft. This research was approved by the Hamilton Integrated Research Ethics Board (project number: 14–334).

### Study 1: Face validity

#### Participants and process

A purposive sampling strategy was used (rather than a representative sampling strategy) to optimise variation in participants’ professional roles and jurisdictions [12]. To this end, a list of international health systems conference attendees served as the population from which to draw samples of candidate participants. This population was complemented by recommendations from the AGREE-HS Research Team members. The candidates were categorised based on WHO region of origin (i.e. Africa, Americas, Eastern Mediterranean, Europe, South-East Asia, Western Pacific) [13]. Three waves of recruitment enlisted the participation of individuals that were randomly selected from each of the six WHO regions; each wave included a new batch of candidate participants.

Selected individuals were sent an email invitation that provided a description of the study and included a link to the AGREE-HS Draft, a link to the survey and an option to opt-out of the study. Once participants linked to the survey platform and their consent was obtained, each survey generated an anonymous numeric code to ensure participants’ confidentiality. Participants were asked to review (but not apply) the AGREE-HS Draft. They were then asked to complete a survey regarding their perceptions of the tool.

#### AGREE-HS draft

The AGREE-HS Draft comprised five quality items (topic, participants, methods, recommendations, and implementability), each with a description and series of criteria. Every item was answered using a 7-point response scale (1 = lowest quality to 7 = highest quality), with higher scores indicating more of the criteria being met. The AGREE-HS Draft concluded with two overall assessment questions, as follows: (1) rate the overall quality of the HSG (1 = lowest quality to 7 = highest quality) and (2) do you recommend this HSG for use? (yes, yes with modifications, no).

#### Face validity survey

The survey was hosted on the LimeSurvey platform™ and included 26 questions, targeting the following issues:

- Clarity of the Overview section (1 = strongly disagree to 7 = strongly agree)
- Perceptions about the User Manual (1 = strongly disagree to 7 = strongly agree)
- Clarity of content for each of the five quality items and two overall assessment questions
- Perceptions about the structure of the tool (1 = strongly disagree to 7 = strongly agree)
- Ranking of importance of the five quality items and usefulness of the two overall assessment questions
- Usefulness and feasibility of using the tool for HSG development, reporting and appraisal purposes (yes – no)
- Participants’ intent to use the tool for HSG development, reporting and appraisal purposes (1 = strongly disagree to 7 = strongly agree)

Participants were also encouraged to provide written feedback.

#### Analyses

Descriptive statistics (means and standard deviations) were calculated. While no specific differences between regions or roles were hypothesised, the number of completed responses was too low to explore potential differences. The quantitative data and descriptive feedback were used to refine wording, structure and the User Manual instructions. These revisions led to the development of the AGREE-HS Final Draft.

### Study 2: Usability and testing

#### Participants and process

A purposive sample of 10 graduate students from McMaster University (Canada) was recruited to participate in Study 2. To optimise variation in perspective, trainees were recruited from three graduate programmes (i.e. health policy, health research methodology and public health). Limited experience and exposure to HSG was sought to enable a better estimate of the usability of the tool among its expected users (e.g. junior staff and researchers). Participants attended a practical training session to learn about the tool and how to apply it. They then completed an appraisal for each HSG document that was allocated to them, using the AGREE-HS Final Draft. Each document was appraised by two participants (the results of the appraisals will be available in a separate publication). Afterwards, participants completed the Usability survey. All materials and surveys were accessible through the LimeSurvey platform™.

#### HSG

A selection of publicly available HSG was sought for inclusion in this study. HSG were eligible if they met the following criteria:

- Addressed an issue related to health system governance, financial or delivery arrangements, or the implementation strategies needed to get the right programmes and services to those who need them
- Included recommendations or guidance statements
- Considered published scientific research and/or local, national or regional data
- Developed by committees from governmental or non-governmental organisations
- Created for health policy-makers, health systems stakeholders or researchers
- Published in the English language
- Published between January 2012–March 2017

Databases and directories used to search for HSG were WHO, the National Institute for Health and Clinical Excellence (NICE), Health Systems Evidence (healthsystemsevidence.org) and a directory of HSG curated by members of the AGREE-HS Research Team for another project. Overall, 85 HSG documents met the inclusion criteria.

#### AGREE-HS final draft

The AGREE-HS Final Draft was used to appraise the 85 included HSG documents. It comprised the five quality items included in the previous iteration of the tool, but with editorial modifications and wording refinements made in response to feedback received during Study 1. It concluded with three overall assessment questions:

- Rate the overall quality of this health systems guidance (1 = lowest quality to 7 = highest quality)
- I would recommend this health systems guidance for use in the appropriate context (yes, yes with modifications, no)
- I would recommend this health systems guidance for use in my context (yes, yes with modifications, no)

#### Usability survey

The survey was hosted on the LimeSurvey platform™ and consisted of 20 questions, targeting the following issues:

- Clarity, helpfulness and completeness of the User Manual (1 = strongly disagree to 7 = strongly agree)
- Appropriateness of level of detail (too much, right amount, not enough)
- Overall ease of use and confidence in using the tool (1 = strongly disagree to 7 = strongly agree)
- Understandability and usefulness of applying each of the five quality items (1 = strongly disagree to 7 = strongly agree)
- Usefulness of the overall assessment questions (1 = strongly disagree to 7 = strongly agree)
- Usefulness of the tool for development, reporting and appraisal purposes and determining whether or not to use an HSG document (1 = strongly disagree to 7 = strongly agree)
- Intention to use the tool for development, reporting and appraisal purposes (1 = strongly disagree to 7 = strongly agree)

Participants were invited to provide written feedback upon completion of each section of the survey.

#### Analyses

##### Usability survey

Descriptive statistics (means and standard deviations) were calculated. Data were used to make final changes to the tool.

##### Measurement properties

To measure its internal consistency, Cronbach’s alpha was calculated with the five quality items [12]. While the study was not specifically powered for this purpose, intraclass correlations (two-way random model) were calculated between the ratings for each item to explore inter-rater reliability [12]. As a surrogate for criterion validation, due to the absence of an established reference standard, a multiple linear regression analysis was calculated to determine which of the five quality items were significant predictors of the overall quality assessment [12].

## Results

### Study 1: Face validity

#### Participants

Completed surveys were received from 30 participants, with representation from each of the WHO regions (18 countries in total) (Table 1). The majority of respondents were health systems researchers (63%) who had at least some experience with developing (83%) or implementing HSG (80%).

**Table 1:** Study 1 (face validity) participant demographics

Characteristic		Frequency
WHO Region	Countries represented	
Africa	Ghana	1
	Nigeria	1
Americas	Argentina	1
	Canada	3
	Colombia	1
	Peru	1
Eastern Mediterranean	Sudan	1
Europe	Netherlands	4
	Switzerland	1
	United Kingdom	2
South-East Asia	Bangladesh	2
	India	1
	Indonesia	3
	Thailand	1
Western Pacific	Australia	1
	China	4
	Papua New Guinea	1
	Philippines	1
Age, years		
< 30		0
30–39		12/30 (40%)
40–49		9/30 (30%)
50–59		5/30 (17%)
60–69		4/30 (13%)
70 or older		0
Professional role/position (multiple responses permitted)		
Health policy-maker		5/30 (17%)
Healthcare provider		2/30 (7%)
Healthcare manager or administrator		7/30 (23%)
Health systems researcher		19/30 (63%)
Student		1/30 (3%)
Methodologist		1/30 (3%)
Other (advisor, programme manager)		7/30 (23%)
EXPERIENCE: Developing HSG		
No experience		5/30 (17%)
Some experience		18/30 (60%)
Experienced		6/30 (20%)
Very experienced		1/30 (3%)
EXPERIENCE: Applying/Implementing HSG		
No experience		6/30 (20%)
Some experience		16/30 (53%)
Experienced		7/30 (23%)
Very experienced		1/30 (3%)

*HSG* health systems guidance

#### Survey results

Table 2 provides an overview of the survey results. Favourable ratings were found for the components designed to assess the AGREE-HS Draft Overview section (mean 5.8 to 5.9), User Manual (mean 5.3 to 6.2), item content (mean 5.6 to 6.0), item structure (mean 5.3 to 5.8), and the assessment questions (mean 5.5 to 5.7). The majority of respondents indicated that the AGREE-HS Draft would be useful to assist in how HSG should be developed, direct what information should be reported in an HSG document and guide the appraisal process (73%, 70% and 90%, respectively). Given the context in which they work, the majority of the respondents also indicated that the AGREE-HS Draft would be feasible to use in HSG development, to direct its reporting and to guide appraisal (60%, 67% and 80%, respectively). Finally, the participants indicated they would use the tool to assist in their HSG development (mean 5.5), reporting (mean 5.7) and appraisal (mean 5.6) activities.

**Table 2:** Study 1 (face validity) survey results

Component	Mean^a^	SD
AGREE-HS Overview Section		
The ‘Background’ section clearly describes the purpose of the AGREE-HS	5.9	0.7
The ‘AGREE-HS Items and Criteria’ section clearly describes how the AGREE-HS is structured	5.7	0.9
The ‘Application of the AGREE-HS’ section clearly describes who the AGREE-HS is intended for	5.9	0.8
User Manual Instructions		
How to use the 7-point response scale is clear	6.1	0.8
How to calculate the overall score is clear	6.1	0.8
How to interpret the overall score is clear	5.2	1.4
Overall, the instructions are clear	5.6	0.9
Based on the instructions, I feel confident about applying the AGREE-HS tool	5.4	1.2
AGREE-HS Item Content		
The content presented on the Topic item page is clear	5.8	1.0
The content presented on the Participants item page is clear	5.9	1.0
The content presented on the Methods item page is clear	5.6	1.1
The content presented on the Recommendations item page is clear	5.9	1.2
The content presented on the Implementability item page is clear	5.8	1.2
AGREE-HS Item Structure		
The structure of the item pages is comprehensive	5.7	1.0
The structure of the item pages is logical	5.8	0.8
The structure, format and content of the item pages will enable users to appraise the item correctly	5.6	1.0
The level of detail provided on the item pages is appropriate	5.3	1.0
AGREE-HS Assessment Questions		
*Rate the overall quality of the HSG*		
This question is clear	5.5	1.1
This question is useful to include	5.6	1.0
*Do you recommend this HSG for use?*		
This question is clear	5.6	1.1
This question is useful to include	5.7	1.2

^a^Scores are based on a 7-point scale (1 = strongly disagree and 7 = strongly agree)

*HSG* health systems guidance

#### Descriptive feedback

Extensive feedback was provided by participants about ways to improve the clarity of the AGREE-HS Draft. Key themes included the need to include minimum thresholds or benchmarks to inform interpretation of the quality scores, advice on the skills or experience required by individuals before they engage in applying the tool, and provision of editorial modifications and examples to clarify concepts. To prepare for Study 2, only editorial refinements were made to the AGREE-HS Draft. The key structure, content and response scale were preserved. These minor modifications yielded the AGREE-HS Final Draft.

### Study 2: Usability and testing

#### Participants

Participants were enrolled in health research methodology (*n* = 4), health policy (*n* = 3) and public health (*n* = 3) graduate programmes, and were from a range of countries of origin. Over half of the participants had no experience with developing (70%) or implementing HSG (60%). Participants were between 20 and 50 years old.

#### Survey results

Table 3 provides a summary of the survey results. Favourable ratings were found for components designed to assess the User Manual (mean 5.9 to 6.3), overall assessment questions (mean 5.5 to 6.0), the usefulness of the tool (mean 5.7 to 6.4) and its usability (mean 5.4 to 6.0). Each of the quality items was rated as easy to understand (mean 6.0 to 6.6) and easy to apply (mean 5.5 to 6.6). Participants reported strong intentions to use the AGREE-HS Final Draft for developing and reporting (mean 6.2), appraising (mean 6.6) and determining the suitability of HSG for implementation (mean 5.9).

**Table 3:** Study 2 (usability) survey results

Component	Mean^a^	SD
Instructions		
The AGREE-HS instructions are clear	6.0	0.4
The AGREE-HS instructions are helpful	6.3	0.4
The AGREE-HS instructions are complete	5.9	0.6
Overall Usability		
The AGREE-HS is easy to use	6.0	0.6
I am confident with the ratings I assigned	5.4	0.6
Core Items		
The item, “Topic” is easy *to understand*	6.6	0.5
The item, “Topic” is easy *to apply*	6.6	0.5
The item, “Participants” is easy *to understand*	6.5	0.5
The item, “Participants” is easy *to apply*	6.4	0.6
The item, “Methods” is easy *to understand*	6.2	0.6
The item, “Methods” is easy *to apply*	6.2	0.6
The item, “Recommendations” is easy *to understand*	6.1	0.7
The item, “Recommendations” is easy *to apply*	5.9	0.8
The item, “Implementability” is easy *to understand*	6.0	0.6
The item, “Implementability” is easy *to apply*	5.8	0.9
The AGREE-HS is complete; the items address all key quality components of HSG	5.3	1.4
Overall Assessment Items		
The question, “Rate the overall quality of this health systems guidance” is useful	6.0	1.2
The question, “I would recommend this health systems guidance for use in the appropriate context” is useful	5.5	1.4
Usefulness		
The AGREE-HS would be useful for evaluating HSG	6.4	0.5
The AGREE-HS would be useful for HSG development and reporting	6.3	1.2
The AGREE-HS would be useful for deciding whether or not to implement HSG	5.7	0.6
The AGREE-HS adds value to the HSG enterprise	6.4	0.5

^a^Scores are based on a 7-point scale (1 = strongly disagree and 7 = strongly agree)

*HSG* health systems guidance

#### Measurement properties

The internal consistency of the AGREE-HS Final Draft was acceptable (*r* = 0.79), with the Cronbach’s alpha increasing to *r* = 0.85 (i.e. good) with the deletion of the topic item [14]. Intraclass correlations with two raters were 0.15, 0.82, 0.73, 0.63 and 0.48 for the topic, participants, methods, recommendations and implementability items, respectively. The multiple linear regression analysis showed that the five quality items could explain 87.3% of the variance of the overall assessment rating. With the exception of the topic item, all other items were significant predictors of the overall assessment rating.

#### Descriptive feedback

As in Study 1, considerable feedback and recommendations were provided by participants, primarily editorial in nature, to improve the clarity of concepts. In addition, participants indicated there would be value in having additional resources available for individuals less familiar with the tool, to improve confidence in its application.

## Discussion

### Actions and refinements

Based on the results of the two studies, five substantive issues emerged which required action and refinement to create the final draft of the tool, called the AGREE-HS (Version 1).

#### Experience and training

As with any new tool, applying the AGREE-HS becomes easier with experience. In Study 2, while participants indicated that the AGREE-HS Final Draft’s five quality items were easy to understand and easy to apply (mean ≥ 5.8), ratings were somewhat lower when they were asked about their confidence in the ratings they assigned (mean 5.4). The latter finding was also seen in Study 1 and, while no quantitative data exist, anecdotal data confirm this experience when the AGREE II tool for CPGs was released [15]. As part of the overall AGREE research programme, and in response to these data, the AGREE Enterprise website will host the AGREE-HS (Version 1) in addition to a range of resources, tutorials and examples to optimise its application [9]. This is particularly important for supporting the development, reporting and appraisal of the topic item, which may be more context specific and nuanced than the other four quality items.

#### For which HSGs?

Some participants from Study 2 questioned whether some of the documents they were appraising were eligible as HSG. We ensured that formal inclusion criteria were set and applied by two independent raters; thus, we have confidence in the documents that were used in the study. The AGREE-HS Final Draft quality items and criteria identified the explicit information, steps and considerations that should occur in a high quality HSG development process and that should be reported in a high quality HSG document. That some documents may be formatted differently, fail to report information, or be called by another name, does not make them any less of an HSG document; rather, they may be of poor quality. This point has been discussed in the AGREE-HS (Version 1) User Manual. Additionally, an AGREE-HS Reporting Checklist, which can be used as a resource by the HSG community to increase capacity and skills, has been developed by the Research Team (Additional file 1).

#### Modifications to items

In both studies, the five quality items were rated as clear, easy to understand and easy to apply. The internal consistency of the items is acceptable and, while not designed specifically as a reliability study, the results from Study 2 showed the inter-rater reliability of the items to be moderate to excellent, with the exception of one item, topic. Indeed, the topic item also did not predict the overall quality assessment. For now, this item has been retained given that this concept has been supported in all other components of this programme of research. Its poorer measurement properties may be attributed to it being more context and experience dependent. That is, AGREE-HS users may require more experience and understanding of a particular HSG document to be able to confidently apply the topic item; therefore, refinement of this item was made. Additionally, the overall assessment question, ‘rate the overall quality of this health systems guidance’, has been removed in the AGREE-HS (Version 1). In its place, users are instructed to calculate an overall quality score from the five quality items only, as this is viewed as a more robust, reliable and valid assessment of quality.

#### Scoring instructions

The AGREE-HS tool has been designed to facilitate the development, reporting and appraisal of HSG documents. AGREE-HS quality scores can be used to categorise HSG (see ‘Thresholds and Benchmarking’ below) or to facilitate discussion and build capacity among users when their scores do not align. To this end, two scoring strategies are offered. In situations where the preservation of the diverse perspectives across appraisers is prioritised, individual rating scores for each quality item (and total item scores) can be averaged, and measures of variance can be derived to reflect this diversity. Alternatively, users may wish to discuss scores in a group setting to create a consensus score for the individual items. While different in purpose, each option is a legitimate scoring strategy. Users should be explicit about scoring methods used and make decisions before the scoring process begins, given that the choice of strategy may impact the conclusions drawn and the decisions made. These suggestions are described in the AGREE-HS (Version 1).

#### Thresholds and benchmarking

Participants in both studies indicated the need for additional direction about how item and overall quality scores should be interpreted. While more research is required to create empirically derived benchmarks to define high and low quality HSG, we have offered examples of how the data can be interpreted. In situations where classification systems are preferred, users could perform a tertile split of the overall quality scores of the candidate HSG documents being considered and then categorise them within each tertile as higher, moderate or lower quality documents. Alternatively, users may establish threshold scores a priori through consensus. For example, HSG documents with mean total scores above 5.0 may be defined as high quality, mean total scores below 3.0 may be defined as low quality, and all else defined as medium quality. Finally, users may determine, again through a priori consensus, that a certain item is more relevant and important for their decision-making purposes. As a consequence, they may primarily consider that particular item, determine a threshold, and use the item’s threshold to differentiate between high and low quality HSG. For example, applying the AGREE II to evaluate CPGs, some users focus on the Rigor of Development domain and only consider CPGs that meet a cut off of 70% in that domain to be of high quality, regardless of the performance in other domains [16]. These examples are included in the AGREE-HS (Version 1).

### Strengths and limitations

A key strength in developing the AGREE-HS (Version 1) was the diversity among the participants with respect to geography/jurisdiction, professional roles and experience with HSG. This diversity is reflected in the membership of the AGREE-HS Research Team, the individuals who participated in the two studies reported here and in the earlier studies [10, 11]. While the absolute number of participants in Study 1 was relatively small, we are reassured that we captured varied opinions by confirming regional representation and professional responsibilities. A second strength of this study was the use of high quality methods to assess face validity and usability, and to conduct preliminary testing of measurement properties.

A limitation to these studies, and the programme of research overall, was that it was almost exclusively conducted within the context of English language HSG and among English language speakers. While French language literature and interviews were considered in earlier stages, we were not consistent in using them throughout the programme of research. Additionally, trainees who appraised the HSG documents had varied level of experience and expertise relating to health policy and systems. However, they attended a training session to learn about the AGREE-HS tool and how to apply it. They were also encouraged to contact the AGREE-HS team if they encountered any problems while completing their assigned appraisals.

### Next steps and future directions

The AGREE-HS (Version 1) is now released to the health systems community. The AGREE Enterprise website provides access to the tool and the AGREE-HS Reporting Checklist, and over time, a range of resources and training materials to optimise the application of the tool [9]. Like the AGREE II [16], we will be developing an interactive training platform and will be encouraging translations of the AGREE-HS (Version 1) into various languages. Through its application and feedback, we hope to gain experience and data to continue to refine the tool. Ongoing assessment of its measurement properties will be important. Moreover, as more methodological and conceptual studies are conducted and are published in the literature, the AGREE-HS (Version 1) must be agile and adapt accordingly.

## Conclusions

Like CPGs, HSG is becoming an increasingly important resource to promote high quality healthcare and strong health systems [2]. HSG documents provide actionable recommendations that can be used by health policy-makers and stakeholders; however, as with any new health innovation, methods to optimise their development and strategies to differentiate between HSG of higher and lower quality are lacking [1]. Our programme of research was designed to address this gap.

Following international standards of measurement design and an integrated knowledge translation approach, we conducted a critical interpretive synthesis to generate HSG quality concepts, developed an initial AGREE-HS prototype, and vetted it with an international group of HSG developers, users, researchers and experts [10, 11]. Using data from these studies, we refined the prototype to create the two drafts of the tool (the AGREE-HS Draft and the AGREE-HS Final Draft), the objects of investigation in the two studies reported here. In Study 1, we recruited participants from each of the WHO regions (18 countries) who provided favourable ratings about the structure, the Overview section, the User Manual and the items of the AGREE-HS Draft. After refinement, the AGREE-HS Final Draft was applied to 85 HSG documents, and usability testing, as well as testing of the tool’s measurement properties, was undertaken (Study 2). The results of Study 2 indicated that the tool is easy to use and that the right amount of instruction is provided to users. The initial measurement properties of the tool were strong and final refinements were made to create the AGREE-HS (Version 1).

The AGREE-HS (Version 1) is a valid and reliable tool for HSG appraisal. It comprises a User Manual, five quality items and two overall assessment questions. The AGREE-HS does not describe a specific operational method for HSG development and reporting. Rather, its quality criteria are meant to serve as a blueprint for HSG development and reporting; an AGREE-HS Reporting Checklist has been created to provide a more user-friendly format of the tool for these purposes (Additional file 1), and is available on the AGREE Enterprise website [9]. The AGREE-HS items, along with their definitions and associated criteria, are available as a supplement to this article (Additional file 2). The AGREE-HS (Version 1) is available in its entirety on the AGREE Enterprise website [9].

## Additional files

Additional file 1 AGREE-HS Reporting Checklist. (DOCX 683 kb)

Additional file 2 AGREE-HS item information. (DOCX 23 kb)

## Abbreviations

AGREE-HS: Appraisal of Guidelines for Research and Evaluation–Health Systems
CPG: clinical practice guideline
HSG: health systems guidance
