# Supplementary material for: Validity and usability testing of a health systems guidance appraisal tool, the AGREE-HS
Source: Health Res Policy Syst. 2018 Jun 20;16:51. doi: 10.1186/s12961-018-0334-9 (PMC6011397; doi:10.1186/s12961-018-0334-9)
Supplement: Supplementary file 1 — AGREE-HS Reporting Checklist. (DOCX 683 kb) [file 12961_2018_334_MOESM1_ESM.docx]

**Additional File 1.** AGREE-HS
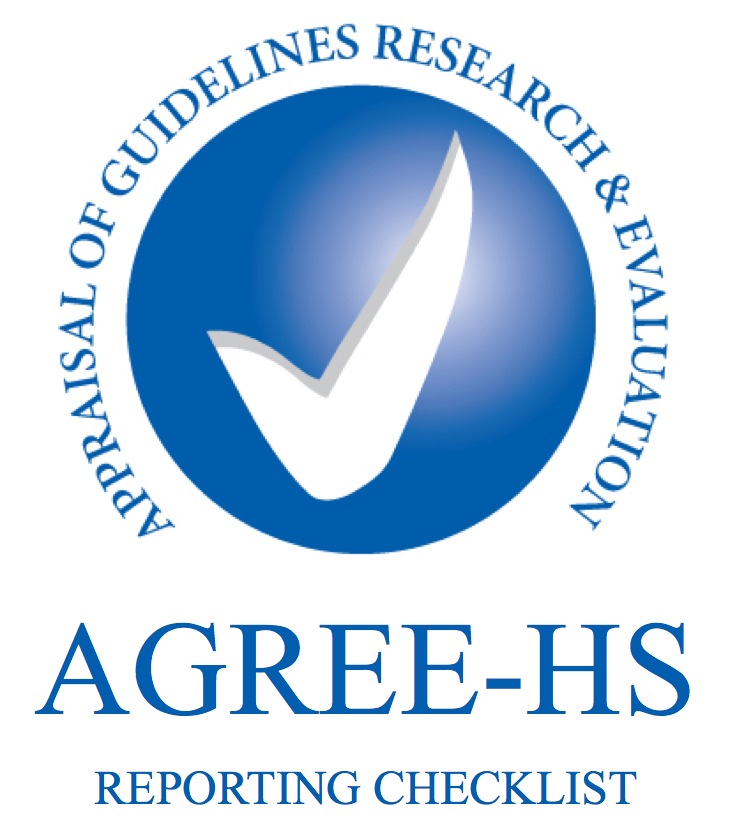
Reporting Checklist

**AGREE- Health Systems (AGREE-HS) Reporting Checklist**

2018

*This checklist is intended to guide the reporting of health systems guidance documents.*

Manuscripts related to the AGREE-HS have been submitted to peer-reviewed journals for publication. Citations will be added here when they are available.

For more information about the AGREE-HS Reporting Checklist, please visit the AGREE Enterprise website at <http://www.agreetrust.org>

| **ITEM DESCRIPTION** | **REPORTING CRITERIA** | **PAGE #** |
| --- | --- | --- |
| ***ITEM 1: TOPIC*** | |  |
| *This item addresses the description of the health system challenge, the causes of the challenge and the priority accorded to it, and relevance of the guidance.* | - The health system challenge is clearly described (i.e., the nature of the challenge; the magnitude, frequency or intensity of the challenge; the populations affected). |  |
|  | - The causes of the health system challenge are clearly described. |  |
|  | - The health system challenge is described in terms of its level of priority in the targeted health system and the affected population; arguments to support the priority classification are provided. |  |
|  | - The guidance is relevant to (i.e., timely in relation to when decisions will be made), and appropriate for, the health system challenge, the system or sub-system needs, the target population(s), and the setting(s) in which they will operate. |  |
| ***ITEM 2: PARTICIPANTS*** | |  |
| *This item addresses the composition of the health systems guidance development team and the management of competing interests and funder influence.* | - - The health systems guidance development team includes members who have an interest or stake in the recommendations (e.g., decision makers, program managers, operational leaders, consumers and members of the public). |  |
|  | - - The health systems guidance development team is multidisciplinary (e.g., political scientists, economists, epidemiologists, methodologists). |  |
|  | - - The health systems guidance development team is multi-sectoral (e.g., primary care, public health and, if appropriate to the challenge, finance and housing). |  |
|  | - - Competing interests of the health systems guidance development team members (e.g., financial, professional), and the strategies used to identify and manage them, are clearly described. |  |
|  | - - Precautions have been taken to avoid or to minimize the influence of a funding agency. |  |
| ***ITEM 3: METHODS*** | |  |
| *This item addresses the use of systematic methods and transparency in reporting; the use of the best available and up-to-date evidence; the consideration of effectiveness and cost-effectiveness of the potential options; and the weighting of benefits and harms in the guidance document.* | - - Systematic and transparent methods were used to identify and review the evidence (e.g., integrated review, scoping review, review of the grey literature, systematic review). |  |
|  | - - The best available and most contextually relevant evidence was considered. |  |
|  | - - The evidence base is current. |  |
|  | - - Evidence of effectiveness of the potential options is clearly described, including descriptions of the contexts in which the options were tested. |  |
|  | - - Evidence of cost and cost-effectiveness of the potential options is described. |  |
|  | - - The weighting of the benefits and harms of the potential options is described. |  |
|  | - - There is a link between the recommendations and evidence. |  |
|  | - - The rationale behind the recommendations is clear. |  |
|  | - - Systematic and transparent methods were used to agree upon the final recommendations (e.g., informal or formal consensus, Delphi method, nominal group methods). |  |
| ***ITEM 4: RECOMMENDATIONS*** | |  |
| *This item addresses the outcomes orientation and comprehensiveness of the guidance; the ethical and equity considerations drawn upon in its development; the details for its operationalization; the sociocultural and political alignment of the guidance; and the updating plan.* | - - The anticipated outcomes of implementing the recommendations are clearly described (including indicators, performance thresholds or targets, and standards to measure them). |  |
|  | - - The recommendations are comprehensive and provide direction to all relevant health system levels (e.g., national, provincial/state), subsystems (e.g., cancer, mental health) and sectors (e.g., primary care, public health). |  |
|  | - - The ethical principles used to develop the recommendations are described. |  |
|  | - - The recommendations promote equity among the target population (e.g., in terms of age, sex, gender, culture, religion, race, sexual orientation). |  |
|  | - - The recommendations’ acceptability to, and alignment with, sociocultural and political interests were considered. |  |
|  | - - The recommendations are easily identifiable, clear, and succinct. |  |
|  | - - The recommendations are actionable and are sufficiently detailed to be operationalized. |  |
|  | - - A plan for updating the recommendations is described. |  |
| ***ITEM 5: IMPLEMENTABILITY*** | |  |
| *This item addresses the barriers and enablers to implementing the recommendations; the cost and resource considerations in implementing the recommendations; the affordability of implementation and anticipated sustainability of outcomes; the flexibility and transferability of the guidance; and the strategies for disseminating the guidance, monitoring its implementation and evaluating its impact.* | - Barriers and enablers to the implementation of the recommendations are described, including factors that are internal (e.g., resources, incentives, administrative structure) and external (e.g., legal system, social system, state of the economy, corruption, beliefs) to the health system. A plan to mitigate barriers and optimize enablers is included. |  |
|  | - Cost and resource considerations for the recommended actions are described (e.g., money, time, infrastructure, equipment, administrative capacity, supplies, staffing, and training). |  |
|  | - The stakeholders’ acceptability of the recommendations is described. |  |
|  | - The affordability of the recommendations, in the context where implementation will take place, is described. |  |
|  | - The anticipated sustainability and requirements to maintain long-term outcomes is described. |  |
|  | - The recommendations are flexible and there is a description of how they can be adapted or tailored for specific contexts in which they will be implemented. |  |
|  | - A description of the degree to which the recommendations are transferable to other similar or different contexts is provided. |  |
|  | - Strategies for disseminating the health systems guidance are described. |  |
|  | - Strategies for assessing the implementation process and the impact of the recommendations are described. |  |
